# Isolation and Purification of Oridonin from the Whole Plant of *Isodon rubescens *by High-Speed Counter-Current Chromatography

**DOI:** 10.3390/molecules16097949

**Published:** 2011-09-14

**Authors:** Fa He, Yuhua Bai, Jing Wang, Jing Wei, ChunYue Yu, Sen Li, Weili Yang, Chenghua Han

**Affiliations:** 1 College of Pharmacy, Harbin University, Daqing 163319, China; Email: hefa8204@163.com (F.H.); wj8859@126.com (J.W.); yuchunyue@163.com (C.Y.Y.); lisen_116@163.com (S.L.); Weiliyang@126.com (W.Y.); hanchenghua@hotmail.com (C.H.); 2 Petrochemical Research Institute Daqing Petrochemical Research Center, Daqing 163714, China; Email: vickyjing@163.com

**Keywords:** *Isodon rubescens*, high-speed counter-current chromatography (HSCCC), oridonin

## Abstract

Semi-preparative high-speed counter-current chromatography (HSCCC) was successfully used for isolation and purification of oridonin from *Isodon rubescens* by using a two-phase-solvent system composed of *n*-hexane-ethyl acetate-methanol-water (2.8:5:2.8:5, v/v/v/v). The targeted compound isolated, collected and purified by HSCCC was analyzed by high performance liquid chromatography (HPLC). A total of 40.6 mg of oridonin with the purity of 73.5% was obtained in less than 100 min from 100 mg of crude *Isodon rubescens* extract. The chemical structure of the compound was identified by IR, ^1^H-NMR and ^13^C-NMR.

## 1. Introduction

*Isodon rubescens *is a plant from the Labiatae family, a perennial herb of the *Isodon *genus, a well-known traditional Chinese medicinal herb, officially listed in the Chinese Pharmacopoeia and widely cultivated in Henan Province, China. It is used in Chinese folk medicine to treat stomach aches, pharyngitis, sore throats, coughs and as an antitumor remedy for the treatment of esophageal and cardiac carcinoma, as the whole plant of *Isodon rubescens *possesses strong anticancer activity [1], Its leaves have been used for the treatment of esophageal cancer for a long time [2]. Oridonin from *Isodon rubescens *was found to have broad spectrum anti-tumor and antibacterial activities *in vitro* and *in vivo* and considered to be a potential new cancer chemoprevention agent [3]. This prompted us to isolate the Oridonin from this plant, and since the conventional chromatography method is tedious and less productive [4,5], we tried to isolate the active anticancer compound from *Isodon rubescens *using high-speed counter-current chromatography (HSCCC).

**Figure 1:**
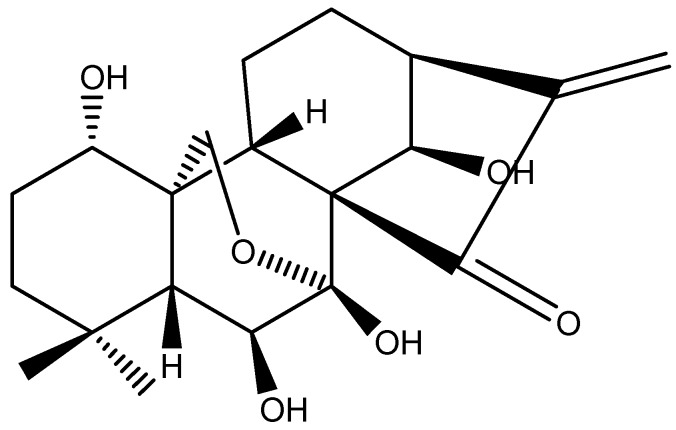
The structure of oridonin.

HSCCC is now accepted as an efficient preparative technique, and widely used for separation and purification of various natural and synthetic products [6], Many natural products have been efficiently separated by HSCCC [7]. HSCCC offers excellent sample recovery and permits directly introduction of crude samples into the column without any preparation, providing relatively pure substances in large amounts. It has been used especially for the analysis and separation of active components from natural products. In the present study, oridonin was obtained from the whole plant of *Isodon rubescens *by HSCCC after optimization of the isolation conditions in terms of solvent system, flow rate and resolution speed. The chemical structure of the isolated compound (Figure 1) was elucidated by IR spectra, ^1^H-NMR and ^13^C-NMR.

## 2. Results and Discussion

### 2.1. Optimization of HPLC Conditions

**Figure 2:**
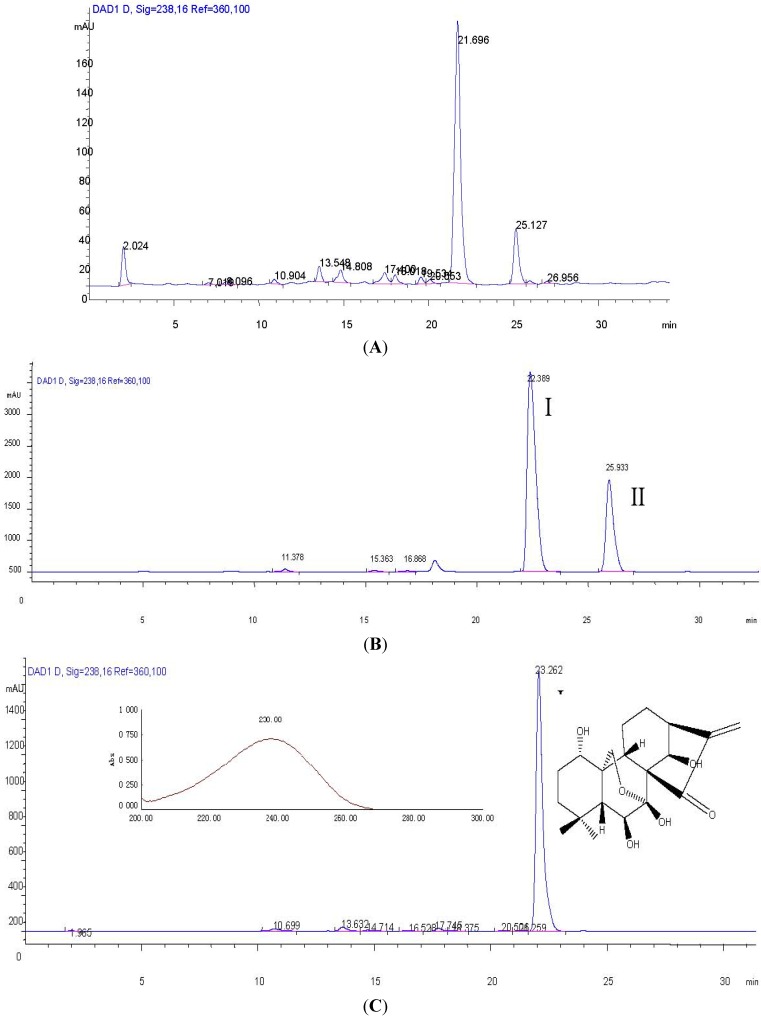
HPLC chromatograms of the crude extract from the whole plant of *Isodon rubescens *and the two targeted compounds purified by HSCCC. Conditions: column: reversed phase Agilent ODS C18 column (250 mm × 4.6 mm I.D., 5 μm); mobile phase: methanol-water formic acid (0.5%) in gradient mode as follows: 0–10 min, 20–40% methanol; 10–20 min, 40–60% methanol; 20–35 min, 60–70% methanol.; flow-rate: 0.8 mL/min; detection wavelength: 238 nm. (**A**) The crude extract from the whole plant of *Isodon rubescens*; (**B**) the shaded area of fraction I separated by HSCCC (Figure 3); (**C**) the pure compound oridonin after PTLC purification.

In order to get good resolution of adjacent peaks within a short analysis time, the optimum HPLC conditions were determined, in the beginning, using various mixtures of water-methanol and acetonitrile-water as mobile phase for the analysis of the crude extract from *Isodon rubescens*, but the separation was not satisfactory, Thus 0.5% formic acid was added to ensure a better separation. the results indicated that when methanol-water formic acid (0.5%) was used as mobile phase in gradient mode (0–10 min, 20–40% methanol; 10–20 min, 40–60% methanol; 20–35 min, 60–70% methanol.), baseline separation of the major peaks was possible. The HPLC chromatogram of the crude extract is shown in Figure 2.

### 2.2. Optimization of HSCCC Conditions

An appropriate two-phase solvent system, which provides an ideal range of the partition ratio (*K*) values for the target compounds, plays an important role in HSCCC separation. However, it is difficult to select a suitable two-phase solvent system and approximately 90% of the optimization work was spent on this. Small *K *values usually result in poor peak resolution, while large *K *values tend to produce excessive sample band broadening [6]. According to the properties of terpenoids, a number of two phase solvent systems [8] based on *n*-hexane-ethyl acetate-methanol-water were tested by changing the volume ratio of the solvents to obtain the optimum conditions that could provide suitable *K *values. The measured *K *values of each compound are summarized in Table 1.

**Table 1:** The K-values of the compounds in different two-phase systems.

Solvent system	K-Value	
	I	II
*n*-hexane-ethyl acetate-methanol-water (1:5;1:5)	2.95	2.53
*n*-hexane-ethyl acetate-methanol-water (2.5:5;2.5:5)	1.02	0.53
*n*-hexane-ethyl acetate-methanol-water (2.8:5;2.8:5)	0.79	0.48
*n*-hexane-ethyl acetate-methanol-water (3.5:5;3.5:5)	0.52	0.28

**Figure 3:**
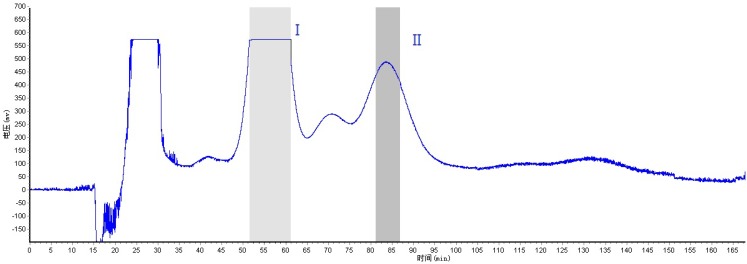
HSCCC chromatogram of the crude extract from the whole plant of *Isodon rubescens.* Two-phase-solvent system: *n*-hexane-ethyl acetate-methanol-water (2.8:5:2.8:5, v/v/v/v); mobile phase: the lower phase; flow-rate: 2 mL/min; revolution speed: 850 rpm; detection wavelength: 254 nm; sample size: 100 mg of crude sample dissolved in 5 mL of the lower phase; separation temperature: 25 °C; retention of the stationary phase: 61%. I, II: collected fractions.

Among them, the solvent system of *n*-hexane-ethyl acetate-methanol-water (1:5:1:5, v/v/v/v) gave too large *K *values and the target compounds needed a long time to elute, resulting in poor resolution. The solvent system of *n*-hexane-ethyl acetate-methanol-water (2.5:5:2.5:5, v/v/v/v) presented suitable *K *values and could be used for sample fractionation, but the HSCCC chromatogram showed that the solvent system of *n*-hexane-ethyl acetate-methanol-water (2.8:5:2.8:5, v/v/v/v) had better resolution, so this two-phase solvent systemwas selected. Apart from a suitable two-phase solvent system, other parameters including flow-rate of the mobile phase and revolution speed of the apparatus, might affect the resolution. The speed of 850 rpm was used invariably in the present study, and the influence of the flow-rate of mobile phase was also investigated. The results indicated that reducing the flow-rate could improve the resolution of stationary phase to some degree, but the chromatogram peaks were spread at the same time, so finally, a flow-rate of 2.0 mL/min was employed in the present study. The crude extract from *Isodon rubescens* was separated and purified under the optimum HSCCC conditions. The HSCCC chromatogram is shown in Figure 3. Compounds I (53.6 mg) and II (13.8 mg) were obtained respectively from 100 mg crude extract, peak I (73.5%) was subsequently purified by PTLC with the solvent of chloroform-acetone 5:6, and 40.6 mg oridonin was obtained; the purity was 97.8%.

## 3. Experimental

### 3.1. Apparatus

The preparative HSCCC instrument employed in the present study was a model TBE-300B high-speed counter-current chromatography system (Shanghai Tauto Biotech Co. Ltd., Shanghai, China) with three multilayer coil separation columns connected in series (I.D. of the tubing = 2.6 mm, total volume = 300 mL) and a 20 mL sample loop. The revolution radius or the distance between the holder axis and central axis of the centrifuge (*R*) was 5 cm, and the *β *values of the multilayer coil varied from 0.5 at internal terminal to 0.8 at the external terminal (*β *= *r*/*R*, where *r *is the distance from the coil to the holder shaft). The revolution speed of the apparatus can be regulated with a speed controller in the range between 0 and 1,000 rpm. An HX1050 constant-temperature circulating bath (Beijing Boyikang Lab Instrument Company, Beijing, China) was used to control the separation temperature. An ÄKTA Prime system (GE Healthcare Biosciences AB, Uuppsala, Sweden) was used to pump the two-phase solvent system and perform the UV absorbance measurements. It contains a switch valve and a mixer, which were used for gradient formation. The data were collected with a ZhengdazhidaN2000 chromatography workstation (Hangzhou Puhui Science Apparatus Company, Hangzhou, China). The HPLC equipment was an Agilent 1200 HPLC system (Agilent Teachnology, Santa Clara, CA, USA) including a G1311A QuatPump, a G1315D UV-vis photodiode array detector, a G1329A autosampler, a G1322A degasser and Agilent HPLC workstation. The Nuclear Magnetic Resonance (NMR) spectrometer was a Bruker 400 NMR system (Bruker Company, karlsruhe, Germany).

### 3.2. Reagents and Materials

All organic solvents used for the preparation of crude sample and for HSCCC separation were of analytical grade (Guangcheng Chemical Factory,Tianjin, China). Methanol and acetonitrile used for HPLC analysis were of chromatographic grade (Yuwang Chemical Factory, Yucheng, China). HPLC-grade water was purified using a Milli-Q system (Millipore, Bedford, MA, USA). Fresh *Isodon rubescens *plants were purchased in June 2010 from the China National Pharmaceutical Group Corporation and air dried. The identity of the plant material was identified by Donghua Wei of Harbin Medical University, and a voucher specimen (NO2010606) is deposited in the Herbarium of the Department of Taxonomy, Harbin Medical University, Daqing, China.

### 3.3. Preparation of the Sample

The dried powder of the whole plants of *Isodon rubescens *(500 g) was ultrasonically extracted three times in 100% methanol (500 mL, 30 min each time). The extracts were combined and concentrated under reduced pressure at 60 °C until the methanol had been removed. The residue was diluted with water (500 mL) and extracted five times successively with water-saturated light petroleum (b.p. 60–90 °C) (500 mL) and ethyl acetate (500 mL); a total of 13.7 g of light petroleum extract and 8.7 g of ethyl acetate extract were obtained. The ethyl acetate extract was further evaporated to dryness under reduced pressure and stored in the refrigerator for subsequent HSCCC separation.

### 3.4. Selection of the Two-Phase-Solvent Systems

The partition coefficient (*K*) is the ratio of solute distributed between the mutually equilibrated two solvent phases. Usually the composition of the two-phase-solvent system was selected according to the partition coefficient of the targeted compounds of crude example. The *K *values were determined by HPLC as follows: crude example (0.5 mg) was dissolved in the aqueous phase of the pre-equilibrated two-phase solvent system (2.5 mL). The solution was separated by HPLC and the peak area was recorded as *A*_1_. Then an equal volume of the organic phase was added to the solution and mixed thoroughly. After the equilibration was established, the aqueous phase was determined by HPLC again and the peak area was recorded as *A*_2_. The partition coefficient (*K*) was obtained by the following equation: *K *= (*A*_1_ − *A*_2_)/*A*_2_.

### 3.5. Preparation of Two-Phase Solvent System and Sample Solution

In the present study, the two-phase-solvent system composed of *n*-hexane-ethyl acetate-methanol-water at volume ratio of 2.8:5:2.8:5 was used for HSCCC separation. Each solvent was added to a separatory funnel and thoroughly equilibrated at room temperature for a whole night. The upper phase and the lower phase were separated and degassed by sonication for 30 min shortly before use. The sample solution for HSCCC separation was prepared by dissolving the dried powder of the crude extract (100 mg) in the lower phase of the two-phase-solvent system *n*-hexane-ethyl acetate-methanol-water (2.8:5:2.8:5, v/v/v/v, 5 mL).

### 3.6. HSCCC Separation Procedure

Firstly, the multiple coiled column was entirely filled with the upper phase of *n*-hexane-ethyl acetate-methanol-water (2.8:5:2.8:5, v/v/v/v) as the stationary phase at a flow-rate of 40 mL/min. Then the apparatus was rotated at 850 rpm, while the lower phase as the mobile phase was pumped into the column by using the ÄKTA Prime system at a flow-rate of 2 mL/min. After the mobile phase front emerged and hydrodynamic equilibrium was established in the column (about 35 min), sample solution (5 mL) containing 100 mg of the crude extract was injected through the injection value by the ÄKTA Prime system. The effluent of the column was continuously monitored with a UV detector at 254 nm and the chromatogram was recorded immediately after the sample injection. The temperature of the apparatus was set at 25 °C. Peak fractions were collected according to the elution profile and evaporated under reduced pressure. The residual was dissolved in methanol for HPLC analysis. The retention of the stationary phase relative to the total column capacity was computed from the volume of the stationary phase collected from the column after the separation was completed.

### 3.7. HPLC Analysis and Identification of HSCCC Peak Fractions

The crude sample and the peak fractions collected from HSCCC were analyzed by HPLC. The analysis was accomplished with an Agilent ODS C_18_ column (250 mm × 4.6 mm I.D., 5 μm) at 25 °C. Acetonitrile-formic acid (0.5%) and water was used as the mobile phase in gradient elution mode as follows: 0–10 min, 20–40% acetonitrile; 10–20 min, 40–60% acetonitrile; 20–35 min, 60–70% acetonitrile. The flow-rate of the mobile phase was 1 mL/min. The effluents were monitored at 238 nm by a photodiode array detector, the UV spectrum of absorbance versus time and wavelength. Identification of the HSCCC peak fractions was performed by IR, ^1^H-NMR and ^13^C-NMR. 

### 3.8. Structure Identification

Colorless needle-like crystals, m p 247.8–248.2 °C. IR (KBr, cm^−1^): 3433, 3382, 3304, 1711, 1647, 1095, 1080, 1068. ^1^H-NMR (400 M, DMSO-d_6_) δ: 6.04, 5.23 (each 1H, s, H-17), 4.57, 4.32 (each 1H, d, *J = *10 Hz, H-20), 3.33(1H, m, H-1), 4.00 (1H, dd, *J = *8.0 Hz, 9.2 Hz, H-6), 1.50 (3H, s, H-18), 1.47 (3H, s, H-19). ^13^C-NMR (DMSO-d_6_) δ: 208.39 (C-15), 151. 94 (C-16), 119. 14 (C-17), 96.8 (C-7), 73.13. 9 (C-6), 72.37 (C-14), 71.62 (C-1), 62.63 (C-20), 61.50 (C-8), 59.35 (C-5), 52.99 (C-9), 42.60 (C-13), 40.44 (C-10), 38.31 (C-3), 33.25 (C-4), 32.65 (C-18), 29.96 (C-12), 29.27 (C-2), 21.65 (C-19), 19.21(C-11). The ^1^H-NMR and ^13^C-NMR data were compared with the data given in literature [9,10], and the compound thus identified as oridonin.

## 4. Conclusions

HSCCC was successfully used for the simultaneous isolation and purification of oridonin from the traditional Chinese medicinal herb *Isodon rubescens* With a pair of two-phase solvent systems composed of *n*-hexane-ethyl acetate-methanol-water (2.8:5:2.8:5, v/v/v/v ), about a 100 mg amount of the crude extract was resolved during 1 h separation for rapid pre-concentration yielding oridonin with the purity of 97.8%, which can be used as reference substances for chromatography or for bioactivity studies. The structure identification was carried out by IR, ^1^H-NMR and ^13^C-NMR. The result shows that HSCCC is a feasible, economical, and efficient technique for rapid preparative isolation of complicated natural products, with higher purity and yield than conventional methods.The method established in this research could be applied in the separation of other natural products.

## References

[1] Sartippour M.R., Seeram N.P., Heber D., Hardy M., Norris A., Lu Q., Zhang L., Lu M., Rao J.Y., Brooks M.N. (2005). Rabddosia rubescens inhibits breast cancer growth and angiogenesis. Int. J. Oncol..

[2] China Pharmacopoeia Committee (2010). Pharmacopoeia of the People’s Republic of China.

[3] Sun H., Huang S., Han Q. (2006). Diterpenoids from *isodon* species and their bioactivity. Nat. Prod. Rep..

[4] Liu L.M., Chen L., Wang R.H., Wu P. (2001). Studies on chemical constituents of Qinpi. Zhong Cao Yao.

[5] Liu L.M., Wang R.H., Chen L., Wu P., Wang L. (2003). Study on chemical constituents of bark of *Fraxinus rhynchophylla*. Zhong Cao Yao.

[6] Ito Y. (2005). Golden rules and pitfalls in selecting optimum conditions for high-speed counter-current chromatography. J. Chromatogr. A.

[7] Zhao H.Y., Jiang J.G. (2010). Application of chromatography technology in the separation of active components from nature derived drugs. Mini Rev. Med. Chem..

[8] Oka F., Oka H., Ito Y. (1991). Systematic search for suitable two-phase solvent systems for high-speed countercurrent chromatography. J. Chromatogr..

[9] Fujita E. (1970). Terpenoids, part XV. Structure and absolute configuration of of oridonin isolated from *Isodon japonicus* and *Isodon trichocarpus*. J. Chem. Soc. (C).

[10] Sun H.D., Lin Z.W., Qin C.Q., Chao J.H., Zhao Q.Z. (1981). Studies on the chemical constituents of antitumor plant *Rabdosia rubescens (Hemsl.) Hara*. Yunnan Zhi Wu Yan Jiu.

